# Association of Plasma Markers of Alzheimer’s Disease, Neurodegeneration, and Neuroinflammation with the Choroid Plexus Integrity in Aging

**DOI:** 10.14336/AD.2023.1226

**Published:** 2024-10-01

**Authors:** Mustapha Bouhrara, Keenan A. Walker, Joseph S. R. Alisch, Zhaoyuan Gong, Caio H. Mazucanti, Alexandria Lewis, Abhay R. Moghekar, Lisa Turek, Victoria Collingham, Nader Shehadeh, Giovanna Fantoni, Mary Kaileh, Christopher M. Bergeron, Jan Bergeron, Susan M. Resnick, Josephine M. Egan

**Affiliations:** ^1^Laboratory of Clinical Investigation, National Institute on Aging, National Institutes of Health, Baltimore, MD 21224, USA.; ^2^Laboratory of Behavioral Neuroscience, National Institute on Aging, National Institutes of Health, Baltimore, MD 21224, USA.; ^3^Johns Hopkins University School of Medicine, Baltimore, 21224 MD, USA.; ^4^Clinical Research Core, Baltimore, MD 21224, USA.

**Keywords:** choroid plexus, Alzheimer’s disease;, neuroinflammation, aging, quantitative MRI

## Abstract

The choroid plexus (CP) is a vital brain structure essential for cerebrospinal fluid (CSF) production. Moreover, alterations in the CP’s structure and function are implicated in molecular conditions and neuropathologies including multiple sclerosis, Alzheimer’s disease, and stroke. Our goal is to provide the first characterization of the association between variation in the CP microstructure and macrostructure/volume using advanced magnetic resonance imaging (MRI) methodology, and blood-based biomarkers of Alzheimer's disease (Aß_42/40_ ratio; pTau181), neuroinflammation and neuronal injury (GFAP; NfL). We hypothesized that plasma biomarkers of brain pathology are associated with disordered CP structure. Moreover, since cerebral microstructural changes can precede macrostructural changes, we also conjecture that these differences would be evident in the CP microstructural integrity. Our cross-sectional study was conducted on a cohort of 108 well-characterized individuals, spanning 22-94 years of age, after excluding participants with cognitive impairments and non-exploitable MR imaging data. Established automated segmentation methods were used to identify the CP volume/macrostructure using structural MR images, while the microstructural integrity of the CP was assessed using our advanced quantitative high-resolution MR imaging of longitudinal and transverse relaxation times (*T*_1_ and *T*_2_). After adjusting for relevant covariates, positive associations were observed between pTau181, NfL and GFAP and all MRI metrics. These associations reached significance (*p*<0.05) except for CP volume *vs*. pTau181 (*p*=0.14), CP volume *vs*. NfL (*p*=0.35), and T_2_ *vs.* NFL (*p*=0.07). Further, negative associations between Aß_42/40_ and all MRI metrics were observed but reached significance only for Aß_42/40_ *vs*. T_2_ (*p*=0.04). These novel findings demonstrate that reduced CP macrostructural and microstructural integrity is positively associated with blood-based biomarkers of AD pathology, neurodegeneration/neuroinflammation and neurodegeneration. Degradation of the CP structure may co-occur with AD pathology and neuroinflammation ahead of clinically detectable cognitive impairment, making the CP a potential structure of interest for early disease detection or treatment monitoring.

## INTRODUCTION

The brain has barriers with selective permeability that regulate transport both in and out of the brain. The blood-brain barrier (BBB) spans the vast majority of the brain vasculature and controls blood-brain transport. The choroid plexus (CP), present in all four ventricles, is another barrier that additionally provides an interface between cerebrospinal fluid (CSF) and blood-CSF barrier (BCSFB). In short, the CP is composed of cuboidal epithelial cells (CPEs) containing microvilli on the apical surface that encase fenestrated capillaries and a connective tissue matrix layer [1]. Tight junctions form the continuous intercellular barrier between CPEs and are the only element that constitutes the BCSFB. Historically CPEs have been mostly studied from the viewpoint of CSF production, but in more recent times the role of CPEs in secreting factors into the CSF that are necessary for maintaining brain health is becoming more appreciated: these factors include transthyretin as the sole transporter of thyroid hormone into brain, insulin and insulin-like growth factors -I and -II, oxytocin, transforming growth factor-β, and klotho to name but a few [1, 2]. Furthermore, CP allows for the passage of nutrients such as glucose and ketones, proteins and solutes, and inflammatory cells from the capillaries into and around CPEs and into CSF [3]. It has been shown that the CP plays a role in the pathogenesis of various neurodegenerative and inflammatory diseases such as Alzheimer’s disease (AD) and multiple sclerosis [4-9]. In AD for example, β-amyloid peptide (Aβ) accumulates in the brain because of overproduction and/or inadequate clearance. Aβ uptake into CPEs occurs *via* LRP-1, LRP-2 and p-Gp present on their apical surface membranes, thereby favoring its efflux from CSF to blood. Consequently, any dysfunction of CP may adversely impact Aβ efflux, resulting in its buildup in brain tissue as well as in the CPEs themselves and thereby physically preventing usual function of biochemical pathways necessary for maintaining CPE health and CSF production: CPEs are terminally differentiated cells that are not replaced by mitosis and there are no resident stem cells in the CPE layer. Moreover, studies have shown that accumulation of tau in the CP may increase the oligomerization rate of Aβ and impair tau trafficking [10].

During the aging process, morphological changes occur in the CPEs that likely impact function. They become flattened and disordered, lack microvilli, they develop inclusion bodies such as lipofuscin granules and Biondi rings, while the connective tissue layer becomes thickened. Additionally, brain barriers represent critical interfaces for neuroimmune communication and therefore any weakening of the tight junctions between each CPE at the BCSFB leads to leakage of peripheral immune cells into CSF [4]. Consequently, age-related changes of the CP make the brain more vulnerable to neurodegeneration and inflammation.

While there are so far no clinically useful imaging techniques to interrogate CP and CPE function, magnetic resonance imaging (MRI) is emerging as a powerful tool to measure the integrity of the CP’s structure [11]. Previous MRI-based works have assessed the CP macrostructure using volumetric measures, as well as microstructural integrity using relaxometry, particularly longitudinal and transverse relaxation times (*T*_1_ and *T*_2_) [12-17]. *T*_1_ and *T*_2_ both depend on macromolecular tissue composition as well as water mobility. Thus, observed changes in *T*_1_ and *T*_2_ are directly associated with cerebral microstructural tissue changes, with higher values being indicative of higher cerebral tissue deterioration [18]. These previous studies have shown that the CP volume significantly increases during some disease states as well as normal aging, thus correlating somewhat with the morphological changes [13-17]. Moreover, the CP has increases in *T*_1_ and *T*_2_ during normal aging and obesity, showing increased water mobility that suggests a decrease in microstructural integrity [12, 13, 19]. In a recent MRI-based study [20], Fleischer and colleagues found that, in patients with multiple sclerosis (MS) and in experimental mouse MS models, volumetric alterations in the CP occur in response to neuroinflammatory processes, with increased CP volume as the severity of the MS disease increases. This supports the CP’s role in the regulation of the neuroimmune axis related to brain homeostasis and interaction with the peripheral immune and inflammatory systems. Further, using MRI volumetry, Tadayon and colleagues have shown that larger CP volume is associated with lower levels of CSF proteins associated with AD and Parkinson pathologies including Aβ, phosphorated tau and α-synuclein [21], again illustrating involvement of CP in the clearance of CSF proteins. Notwithstanding the importance of these pioneering studies, the association of markers of AD pathology, neuroinflammation and neurodegeneration with the CP’s macrostructural or microstructural integrity has not yet been established. Such studies will advance our understanding of the cause and consequence of CP structural and functional alterations in aging and age-related neurological diseases.

The current study addressed this question by examining the association of CP macrostructure/volume and CP microstructure (assessed using *T*_1_ or *T*_2_) with plasma biomarkers of AD (Aß_42/40_ ratio; pTau181), neuronal injury (neurofilament light chain [NfL]), and reactive astrogliosis (glial fibrillary acidic protein [GFAP]), a maker of astrocyte-mediated neuro-inflammation. Here, we lay the groundwork in our understanding of CP-specific structural changes related to age-related neurodegeneration.

## MATERIALS AND METHODS

### Participants

Participants are part of the Baltimore Longitudinal Study of Aging (BLSA: IRB# 03-AG-0325) [22, 23] and the Genetic and Epigenetic Signatures of Translational Aging Laboratory Testing (GESTALT: IRB# 15-AG-0063) studies. The goal of BLSA and GESTALT is to evaluate multiple biomarkers related to physical and psychological aging. We note that the inclusion and exclusion criteria for these two studies are essentially identical. Participants underwent testing at the National Institute on Aging's clinical research unit and were excluded if they had metallic implants, neurologic, or significant medical disorders. Further, all participants underwent a battery of cognitive tests and cognitive status was adjudicated through research consensus case conferences were indicated by established screening criteria. Participants with cognitive impairment (i.e., mild cognitive impairment [MCI], dementia, or cognitively impaired non-MCI) were excluded [24]. Cognitively normal status was based on either (i) a Clinical Dementia Rating [25] of zero and/or ≤ 3 errors on the Blessed Information-Memory-Concentration Test, indicating that the participant did not meet the criteria for consensus conference, or (ii) the participant was determined to be cognitively normal based on a thorough review of clinical and neuropsychological data [26]. Though cognitively normal, participants were not excluded on the basis of abnormal AD pathology biomarkers; therefore, the study cohort likely includes participants with preclinical AD.

### MR imaging

For each participant, the imaging protocol for *T_1_* and *T_2_* mapping consists of 3D spoiled gradient recalled echo (SPGR) images acquired with flip angles (FAs) of [2 4 6 8 10 12 14 16 18 20]°, echo time (TE) of 1.37 ms, repetition time (TR) of 5 ms, and acquisition time (AT) of ~5 min, as well as 3D balanced steady state free precession (bSSFP) images acquired with FAs of [2 4 7 11 16 24 32 40 50 60]°, TE of 2.8 ms, TR of 5.8 ms, and AT of ~6 min. The bSSFP images were acquired with radiofrequency excitation pulse phase cycling of 0 or π to account for the off-resonance artifacts [27-30]. Images were acquired with an acquisition matrix of 150 × 130 × 94, voxel size of 1.6 mm × 1.6 mm × 1.6 mm. We used the double-angle method (DAM) to correct for the inhomogeneities in the excitation radio frequency pulses [31]. DAM consisted in acquiring two fast spin-echo images with FAs of 45° and 90°, TE of 102 ms, TR of 3000 ms, acquisition voxel size of 2.6 mm × 2.6 mm × 4 mm, and acquisition time of ~4 min. All images were acquired with field of view (FoV) of 240 mm × 208 mm × 150 mm, and reconstructed to 1 mm × 1 mm × 1 mm. The total acquisition time was ~21 min. All MRI studies were performed were performed on a 3T whole body Philips MRI system (Achieva, Best, The Netherlands), using the internal quadrature body coil for transmission and an eight-channel phased-array head coil for reception, after approval by the MedStar Research Institute and the National Institutes of Health Intramural Ethics Committees. All examinations were performed in compliance with the standards established by the National Institutes of Health Institutional Review Board, and written informed consent was obtained.

### Image processing

CP volume determination: for each participant, corresponding *T_1_*-weighted SPGR images were used. Specifically, the FreeSurfer Aseg Atlas [32] was nonlinearly registered to the SPGR images averaged over all FAs using the cortical reconstruction (*recon-all*) pipeline from the Freesurfer v7.1.1 software [33], and the CP volume was then calculated. This method has been used in several other studies indicating reliable CP segmentation [13-15, 17, 34-36]. All CP volumes were thoroughly examined and corrected manually when needed. The CP volume was normalized to the total cranial volume to account for variations in individual head size.

T_1_ and T_2_ mapping: for each participant, using the FMRIB Software Library (FSL) software [37], all SPGR, bSSFP, and DAM images were linearly registered to the SPGR image acquired at a FA of 8° and the derived transformation matrix was then applied to the original SPGR, bSSFP, and DAM images. Next, a whole-brain T_1_ map was generated from the co-registered SPGR dataset using the DESPOT1 analysis and assuming a single relaxing component using the stochastic regions contraction (SRC) algorithm while correcting for transmit field, B_1_, inhomogeneities [38, 39]. The B_1_ map was generated from the co-registered fast spin-echo using the DAM approach [40]. Further, using these derived T_1_ and B_1_ maps as input parameters, a whole-brain T_2_ map was generated from the co-registered bSSFP dataset using the DESPOT2 analysis and assuming a single relaxing component using SRC [38, 39]. Next, using FreeSurfer, the SPGR image averaged over all FAs was registered using nonlinear registration to FreeSurfer's Aseg atlas and the derived transformation matrix was then applied to the corresponding T_1_ and T_2_ maps. Finally, the mean T_1_ and T_2_ values in the CP region were calculated.

### Plasma biomarkers measurement

Blood for plasma biomarker measurements was collected at the time of 3T MRI scanning. Plasma was separated, aliquoted and stored at -80°C using standardized protocols. EDTA plasma was used to measure Aβ_42_, Aβ_40_, GFAP, NfL using the Quanterix Single Molecule Array (Simoa) Neurology 4-Plex E assay (Quanterix Item: 103670) on the HD-X Instrument (Quanterix Corporation). Phosphorylated tau-181 (pTau181) was measured using the Quanterix Simoa p-Tau181 Assay version 2 (Quanterix Item: 104111) on the HD-X Instrument. All assays were run in duplicate, and the mean value of the duplicate measurements was used for all analyses. Biomarkers derived from BLSA and GESTALT were quantified using the same set of assay kits (identical lot numbers). The intraassay coefficients of variation (CV) as derived from the parent study were 1.9, 2.8, 5.0, 5.1, and 4.4 for measures of Aβ_42_, Aβ_40_, GFAP, NfL and pTau181, respectively.

### Statistical Analyses

Multiple linear regression was used to investigate the regional associations between each plasma biomarker (Aβ_42_/Aβ_40_, pTau181, GFAP, or NfL), as the independent variable, and CP macrostructure (volume) or microstructure (T_1_ or T_2_), as the dependent variable, while accounting for relevant covariates, namely, age, sex, body mass index (BMI), and hypertension status. Plasma biomarkers values were log-transformed to remove the skewness of their distributions. The normality of the data distribution was confirmed using the Shapiro-Wilk test. To facilitate results interpretation, the plasma biomarkers and MR imaging indices were Z-scored. Further, in a secondary analysis, we examined the modifying effect of age on the MRI-plasma relationship using a continuous age × plasma interaction term in the regression model above.

Further, since T_1_ and T_2_ strongly depend on water mobility, concerns may arise due to potential contamination of CP’s T_1_ and T_2_ values from CSF water. To address this concern, we explored the potential effect of partial volume on the association between T_1_ or T_2_ derived in CSF and Aβ_42_/Aβ_40_, GFAP, NfL, or pTau181. CSF T_1_ and T_2_ values were calculated in the ventricular CSF regions and used in a regression analysis against Aβ_42_/Aβ_40_, GFAP, NfL, or pTau181 accounting for the confounding variables described above. CSF maps were generated using FSL FAST segmentation [41].

All analyses were performed using MATLAB (MathWorks, Natick, MA, USA), and using the MATLAB function *fitlm* for the linear regression analyses conducted here.

**Table 1:** Participant demographic characteristics.

Total Sample: N	108
Age (yrs.): mean ± SD *(min - max)*	55.7 ± 20.4 *(22-94)*
*Sex*	
Men: n *(%)*	57 *(52.8%)*
Women: n *(%)*	51 *(47.2%)*
MMSE: mean ± SD *(min - max)*	28.8 ± 1.4 *(25-30)*
BMI (Kg/m^2^): mean ± SD *(min - max)* Lean (BMI < 25)*:* n *(%)* Overweight (25 ≤ BMI < 30)*:* n *(%)* Obese (BMI ≥ 30)*:* n *(%)*	25.8 ± 3.6 *(18.2-35.8)* 52 *(44%)* 50 *(42%)* 16 *(14%)*
Hypertension: n *(%)*	23 *(21.3%)*
*Plasma markers* Aβ_42_/Aβ_40_: mean ± SD *(min - max)*	0.06 ± 0.013 *(0.014-0.1)*
pTau181 (pg/mL): mean ± SD *(min - max)*	2.3 ± 1.3 *(0.82-7.11)*
NfL (pg/mL): mean ± SD *(min - max)*	16.6 ± 12.7 *(2.65-82.5)*
GFAP (pg/mL): mean ± SD *(min - max)*	120.5 ± 69.9 *(37.9-396.1)*

SD, standard deviation; min, minimum; max, maximum; MMSE, Mini-Mental State Examination; BMI, body-mass index; Aβ, amyloid-beta; pTau, phosphorylated tau; NfL, neurofilament light chain; GFAP, glial fibrillary acidic protein.

## RESULTS

After exclusion of nineteen participants with either cognitive impairment or problematic imaging datasets due to motion artifacts, the final study cohort consisted of 108 cognitively unimpaired volunteers spanning the age range comprised between 22 and 94 years (mean ± SD = 55.7 ± 20.4 years), of which 57 were men (57.0 ± 21.3 years) and 51 were women (54.2 ± 19.5 years) (Table 1). Age did not differ significantly between men and women (*p* = 0.48). Finally, as expected, given its wide age range, the study cohort exhibits large ranges of Aβ_42_/Aβ_40_, pTau181, NfL and GFAP level values, leading to a great variability and large distributions in these measures.

**Figure 1.**
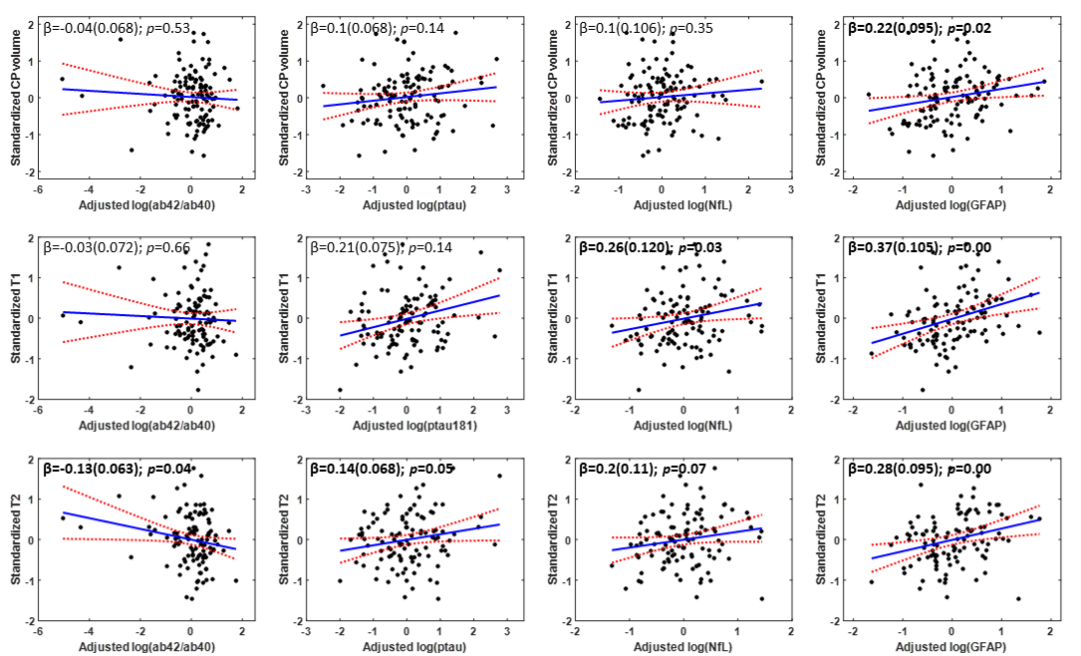
Association of Alzheimer’s disease, neurodegeneration, and neuroinflammation biomarkers with choroid plexus integrity. Regression results (N = 108) for the association of Alzheimer's disease (Aβ42/Aβ40 and pTau181) and neurodegeneration or neuroinflammation (NfL and GFAP) plasma biomarkers with the CP’s volume (top row), T1 (middle row), or T2 (bottom row). For each graph, the line of fit (in blue) and confidence intervals (in red), as well as the regression coefficient, β, (standard error) and p-values, derived from the linear regression model adjusted for age, sex, BMI, and hypertension were displayed. The adjusted response function describes the relationship between the fitted response and MWF, with the other predictors averaged out by averaging the fitted values over the data used in the fit. Bold indicates associations that are statistically significant (p < 0.05).

**Figure 2.**
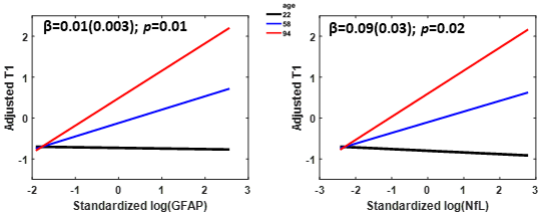
Age-stratified association of GFAP and NfL with choroid plexus integrity. Age-stratified association between CP T1 and GFAP or NfL derived using a linear regression model adjusted for age, sex, BMI, and hypertension status. The age-stratified lines show the adjusted T1 as a function of GFAP or NfL, with age fixed at three specific values of 22 (black line), 58 (blue line), and 94 (red line) corresponding to the minimal, median, and maximal values, respectively. The adjusted response function describes the relationship between the fitted response and plasma biomarker (GFAP or NfL), with the other predictors averaged out by averaging the fitted values over the data used in the fit. The regression coefficient and p-value of the interaction term are displayed.

Figure 1 shows the association of plasma markers of AD (Aβ_42_/Aβ_40_; pTau181), neurodegeneration (NfL), and neuroinflammation (GFAP) with measures of CP volume and microstructural integrity (as measured by T_1_ and T_2_), after adjusting for sex, age, hypertension status, and body mass index (BMI). Visual inspection indicates that higher Aβ_42_/Aβ_40_ values or lower pTau181 values corresponds to lower CP volume, T_1_ or T_2_ values. While the associations between AD biomarkers and CP volume/macrostructure did not reach statistical significance (Table 2), greater plasma pTau181 levels were significantly associated with greater T_1_ or T_2_, indicative of worse microstructural integrity. Lower plasma Aβ_42_/Aβ_40_ levels were associated with lower CP microstructural integrity as measured by T2, but not T_1_ (Table 2). These trends suggest that higher levels of AD pathology are associated with lower CP microstructural, but not macrostructural, integrity. Furthermore, plasma NfL and GFAP were positively associated with measures of CP volume and CP T_1_ and T_2_, suggesting that neuronal injury and reactive astrogliosis are associated with decrements in the macrostructure and microstructure of the CP (Fig. 1). These associations were all statistically significant (Table 3), except for CP volume *vs*. NfL (Table 3), indicating a potential link between elevated plasma biomarkers of neurodegeneration/neuroinflammation and decreased CP’s macrostructural or microstructural integrity. Moreover, our secondary analysis showed that age modified the association of GFAP and NfL with T_1_ (GFAP: interaction-*p* = 0.01, NfL: interaction-*p* = 0.02) in a manner such that higher levels of NfL and GFAP tended to be more strongly associated with reduced CP microstructural integrity (higher T_1_ values) among old and middle-aged subjects than young subjects (Fig. 2). Finally, as expected, statistically significant age effects were observed for all MR metrics evaluated (Tables 2-3). Additionally, the correlations between CP volume, T_1_ or T_2_ and sex, BMI, or hypertension were found to, overall, be statistically nonsignificant (Tables 2-3).

**Figure 3.**
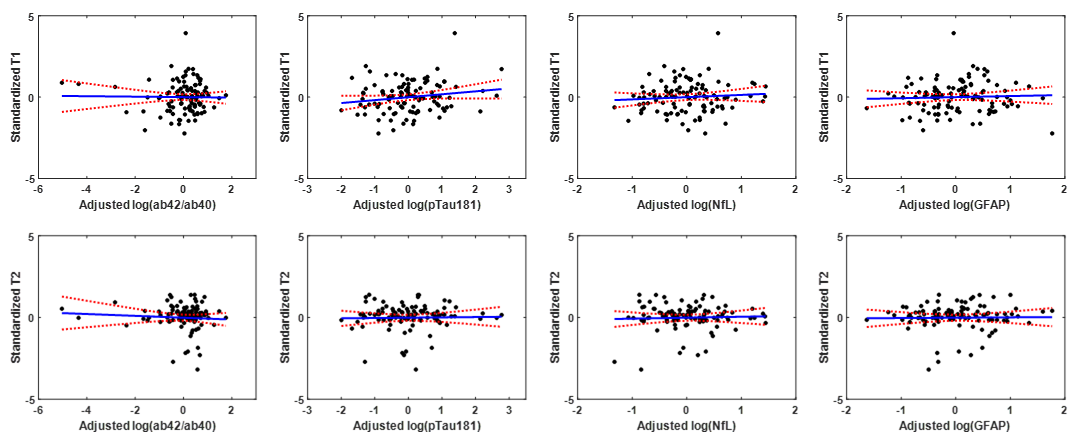
Association of CSF-derived T1 and T2 with plasma biomarkers. Regression results for the relationship between T1 or T2 derived in the ventricular cerebrospinal fluid (CSF), and of Alzheimer's disease (Aβ42/Aβ40 and pTau181) and neurodegeneration or neuroinflammation (NfL and GFAP) plasma biomarkers, adjusted for age, sex, BMI, and hypertension status. All associations were nonsignificant.

Finally, Aβ_42_/Aβ_40_, pTau181, NfL and GFAP showed no association with T_1_ and T_2_ MR parameters when measured in a ventricular CSF region. This lack of association was readily apparent in Fig. 3. Our statistical analysis, adjusting for covariates, indicated that Aβ_42_/Aβ_40_, pTau181, NfL and GFAP exhibited nonsignificant associations (*p* > 0.1) with each of the CSF MR parameters. These results indicate that the associations between CP microstructure and plasma biomarkers described above are not driven by potential contamination from CSF.

## DISCUSSION

Mounting evidence indicates that aging and age-related neurological diseases lead to structural and functional damage to the choroid plexus (CP) that likely has downstream effects on cognitive and functional abilities. However, studies of potential factors influencing degeneration of the CP structure are lacking, particularly before the onset of cognitive impairment. Using plasma biomarkers and state-of-the-art MR techniques to measure the CP volume and its microstructural integrity, the current study provides the first evidence that elevated plasma markers of Alzheimer’s disease (AD) pathology (Aβ_42_/Aβ_40_ and pTau181), neuronal injury (NfL), and neuroinflammation (GFAP) are associated with differences in the CP’s macro- and micro-structure in cognitively normal adults. As expected, since microstructural damages to cerebral tissue precede macrostructural changes, including structural atrophy or hypertrophy, these associations were mostly significant with the MRI measures of microstructural integrity, namely T_1_ and T_2_; these MRI metrics are highly sensitive to early changes in tissue integrity and have been widely used in several investigations of brain maturation and degeneration [42], including complex and small structures such as the brainstem and the CP [43-46]. Therefore, CP microstructural differences can be detected at early stage of diseases, even before noticeable differences in CP functions, providing potential window for early therapeutical interventions.

**Table 2:** Association of Alzheimer’s disease plasma biomarkers with measures of CP volume and microstructural integrity.

	CP volume	T_1_	T_2_
		Aβ_42_/Aβ_40_	
Age	0.03(0.003); *p*<0.01	0.03(0.003); *p*<0.01	0.03(0.003); *p*<0.01
Sex	0.21(0.130); *p*=0.11	0.06(0.142); *p*=0.66	-0.07(0.124); *p*=0.58
BMI	-0.00(0.02); *p*=0.82	0.01(0.02); *p*=0.45	0.00(0.02); *p*=0.86
Hypertension	0.28(0.17); *p*=0.11	0.20(0.19); *p*=0.31	0.08(0.17); *p*=0.63
Aβ_42_/Aβ_40_	-0.04(0.068); *p*=0.53	-0.03(0.072); *p*=0.66	-0.13(0.063); *p*=0.04
		pTau181	
Age	0.03(0.003); *p*<0.01	0.02(0.004); *p*<0.01	0.03(0.003); *p*<0.01
Sex	0.20(0.130); *p*=0.12	0.26(0.137); *p*=0.85	-0.07(0.124); *p*=0.56
BMI	-0.00(0.01); *p*=0.88	0.02(0.02); *p*=0.33	0.00(0.02); *p*=0.72
Hypertension	0.27(0.17); *p*=0.11	0.19(0.18); *p*=0.30	0.02(0.17); *p*=0.87
pTau181	0.1(0.068); *p*=0.14	0.21(0.075); *p*<0.01	0.14(0.068); *p*=0.05

Slope (standard error) and significance, p, of the regression terms incorporated in the multiple linear regression given by: CP volume, T_1_, or T_2_ ~ β_0_ + β_BMI_ × BMI + β_age_ × age + β_sex_ × sex + β_hypertension_ × hypertension + β_AD_ × AD. Bold indicates statistical significance (p < 0.05), and AD stands for Aβ_42_/Aβ_40_ or pTau181.

According primarily to postmortem and animal studies, during normal aging, the CP undergoes several alterations, including morphological changes to the epithelial cells [47-49], calcification [50], increased T helper type 2 response [51], reduced CSF production and clearance [52, 53], iron deposition [54], and decreased secretory capacity of both the endothelial cells of the BBB and the CP epithelial cells of the BCSFB as well as their ability to remove toxic compounds from the brain. Furthermore, it has been demonstrated that the CP, in AD and aging, is also characterized by amyloid-beta (Aβ) deposition that may disrupt various functions of the CP through impeding underlying biochemical pathways [55-58]. In addition, recent studies have shown that accumulation of tau in the CP may increase the oligomerization rate of Aβ_42_ and impair tau trafficking, leading to AD pathology [10]. In line with this seminal work, our results provide evidence indicating that higher levels of Aβ and phosphorylated tau are associated with greater deterioration of the CP integrity, including in individuals without cognitive impairment consistent with evidence that biomarker and brain changes occur years before clinical symptoms. We expect that the extent of this structural deterioration would be even more evident in individuals with AD dementia or other neurodegenerative diseases in the symptomatic stage of the disease process. Our work lays the foundation for these future investigations. Unfortunately, such investigation could not be undertaken in the current study since the BLSA and GESTALT cohorts do exclude participants with cognitive impairments including those with MCI or AD.

Our results suggest that elevated levels of NfL and GFAP are associated with a reduced CP integrity. NfL, increased level of which is an indicator of neuronal injury, is a structural cytoskeleton scaffolding protein that is expressed especially in large quantities by large axons and is sensitive to various neuroinflammatory conditions including multiple sclerosis, Alzheimer’s and Parkinson’s diseases [59-64]. GFAP, a protein that is encoded by the GFAP gene in humans, is expressed by numerous CNS cell types, including astrocytes and ependymal cells during development, and is regarded as a proxy of neuroinflammation. Higher levels of NfL and GFAP have recently been associated with demyelination and axonal degeneration [65]. Our results fit into the hypothesis of a relationship between elevated central inflammation and greater degeneration of the CP structure, especially microstructure, in conjunction with brain barrier dysfunction, in aging. During inflammatory conditions in the CNS, immune cells immigrate into the CNS, and can be detected in both CNS parenchyma and in CSF, initiating molecular and cellular events that ultimately cause CNS tissue degradation [20, 66]. These observations are in line with recent immune-histochemical-based studies identifying the presence of neuroinflammatory processes in the CP [67]. This paradigm is further reinforced with evidence showing that the brain barriers, specifically the BCSFB and the BBB, which represent critical interfaces for neuroimmune communication, are affected by the aging process and correlate with various progressive cellular dysfunctions, including increased permeability of these brain barriers [4]. Taken together, it is therefore conceivable that the decline in the CP’s structural integrity would be affected, at least in part, by these neuroinflammatory processes. Nevertheless, this paradigm requires further investigation, once again with our results providing a basis to these studies.

A strength of the current study is the participant wide age range, which allowed us to determine how age modified the relationship between plasma biomarkers and CP integrity. We found that age appears to modify the relationship between these plasma markers and CP integrity, indicating that lower CP structural integrity tended to be associated with higher levels of AD pathology, neurodegeneration and neuroinflammation among old and middle-aged subjects. Nevertheless, this association achieved significance only for T_1_ *vs*. GFAP or NfL, motivating further studies with larger samples, including longitudinal investigations, to further establish this potential relationship.

**Table 3:** Association of neurodegeneration and neuroinflammation plasma biomarkers and measures of CP volume and microstructural integrity.

	CP volume	T_1_	T_2_
		NfL	
Age	0.03(0.005); *p*<0.01	0.02(0.006); *p*<0.01	0.03(0.005); *p*<0.01
Sex	0.23(0.130); *p*=0.12	0.10(0.139); *p*=0.46	-0.02(0.125); *p*=0.87
BMI	-0.00(0.02); *p*=0.88	0.03(0.02); *p*=0.17	0.00(0.02); *p*=0.46
Hypertension	0.26(0.17); *p*=0.13	0.22(0.19); *p*=0.25	0.04(0.17); *p*=0.78
NfL	0.1(0.106); *p*=0.35	0.26(0.12); *p*=0.03	0.2(0.11); *p*=0.07
		GFAP	
Age	0.02(0.004); *p*<0.01	0.02(0.005); *p*<0.01	0.03(0.005); *p*<0.01
Sex	0.26(0.130); *p*=0.04	0.14(0.135); *p*=0.31	0.01(0.123); *p*=0.95
BMI	-0.00(0.01); *p*=0.91	0.02(0.02); *p*=0.41	0.00(0.02); *p*=0.80
Hypertension	0.27(0.16); *p*=0.10	0.24(0.18); *p*=0.19	0.06(0.16); *p*=0.71
GFAP	0.22(0.095); *p*=0.02	0.37(0.105); *p*<0.01	0.28(0.095); *p*<0.01

Slope (standard error) and significance, *p*, of the regression terms incorporated in the multiple linear regression given by: CP volume (CPV), *T_1_*, or *T_2_*~ *β_0_* + *β_BMI_* × BMI + *β_age_* × age + *β_sex_* × sex + *β_hypertension_* × hypertension + *β_GFAP/NfL_* × GFAP/NfL. Bold indicates statistical significance (*p* < 0.05).

Previous research has predominantly centered on the morphological changes of the CP and their correlation with clinical outcomes. Studies have found a significant increase in CP volume among individuals across different conditions including schizophrenia, bipolar disorder, Parkinson’s disease, and AD as compared to healthy controls. This enlargement was linked to more severe symptoms, cognitive impairments, and neurobiological changes [17, 68, 69]. Another study investigating CP characteristics in humans with multiple sclerosis (MS) and mouse models found a strong association between increased CP volume and ongoing neuroinflammation and clinical disability [20]. Furthermore, a retrospective study focusing on CP inflammation's impact on MS progression revealed that MS patients had notably larger CP volumes and elevated CP pseudo-T2 (pT2) levels at baseline compared to healthy individuals [70]. Significantly, this baseline CP pT2 was correlated with the progression of clinical disability in follow-ups, even after considering other factors that contribute to disability progression. These findings underscore the clinical relevance of CP changes in the progression and management of these neurological disorders. Nevertheless, the association between changes in CP microstructure and changes in cognition, including in normative aging, remains to be established; this represents a future direction of our work.

Although we examined a relatively large cohort and used advanced MR methodology, our study has limitations. Our dataset is cross-sectional so that the observed trends in the CP microstructure or volume with plasma biomarkers of AD, neuronal injury and inflammation require further validation using longitudinal studies. Such work, motivated by the present results, is underway and will allow investigation of the temporal sequence of change in CP macrostructure and microstructure in relation to AD and neurodegeneration biomarkers. In addition, while our results indicate that several of the relationships between the MRI measures and plasma biomarkers have reached statistical significance, given the exploratory nature of this study, modest sample size, and the correlation among predictor variables and among outcome variables, multiple comparison correction was not applied. We believe therefore that application of Bonferroni or FDR correction for this analysis would be overly conservative and (for the aforementioned reasons) would likely inflate our Type II error rate. Therefore, in our work, we considered that a nominal p < 0.05 as suggestive of a significant association. We note, however, that investigations on larger study cohorts are needed to confirm or infirm our observations. The plasma biomarkers used in this study, while broadly accepted as indicators of AD pathology, neurodegenerative, and neuroinflammatory processes, are likely also influenced by non-specific physiological factors and clinical and sub-clinical comorbid disease, much of which lies outside the central nervous system. This is particularly relevant for NfL, which has been shown to be especially susceptible to changes non-neurologic health factors [71]. Further, an outstanding question that cannot be addressed using an observational study design is whether the relationships between greater deterioration of the CP integrity, and increased AD pathology and neuroinflammation are mechanistic or simply associative. Studies have demonstrated that changes in the CP structure occur in different human disease states, including neuroinflammatory conditions, suggesting a key role of the CP in the initiation and/or propagation of inflammatory brain responses [20, 66, 67]. Although the mechanistic link remains unknown, the current study suggests that CP structural damage could be an indicator of AD pathology, neurodegeneration, or neuroinflammation among cognitively unimpaired adults. Furthermore, CSF partial volume effects may bias derived parameter values. However, our analyses indicate that the observed trends of the MR parameters are driven by differences in the CP’s microstructure as all parameters derived in the CSF exhibited nonsignificant associations with plasma biomarkers. Nevertheless, more accurate automated segmentation methods, including the third and fourth ventricles, as well as higher resolution structural images are needed for a better evaluation.
